# Supplementary material for: Acute Exposure to Aerosolized Nanoplastics Modulates Redox-Linked Immune Responses in Human Airway Epithelium
Source: Antioxidants (Basel). 2025 Mar 31;14(4):424. doi: 10.3390/antiox14040424 (PMC12024294; doi:10.3390/antiox14040424)
Supplement: Supplementary file 1 [file antioxidants-14-00424-s001.zip › antioxidants-3482170-supplementary.pdf]

Table S1. Quantification of human cytokines and chemokines from conditioned cell culture media by custom multiplex ELISA-based Q-Plex™ technology.

Cytokines/Chemokines	Protein Quantification (pg/mL)
CTACK	1.67
hCXCL-5	1.16
hGMCSF	0.94
hHGF	0.87
hIL-12p40	0.80
IL-33	0.54
MCP3	0.96
hMIP-1α	4.94*
hMIP-1β	4.80
hEotaxin-3	1.00
IL-21	0.16****
hIL-1α	0.94
hIL-1β	0.63
hIL-2	0.54*
hIL-4	0.63
hIL-5	1.96
hIL-6	1.02
hIL-8	0.96

hIL-10	1.53
hIL-12p70	0.59
hIL-13	0.74
hIL-15	0.10*
hIL-17	1.05
hIL-23	1.84
hIFNy	0.78
hTNFβ	1.18
hTNFβ	1.35
hEotaxin	0.83
hGROα	1.17
hI-309	1.10
hIP-10 (CXCL10)	0.81*
hMCP-1	1.04
hMCP-2	1.21
hRANTES	1.36
hTARC	0.63
hTGFβ	0.826*
